# Closed Gastroschisis

**DOI:** 10.21699/jns.v6i3.599

**Published:** 2017-08-10

**Authors:** Mohammed Abdel-Latif, Mohamed H. Soliman, Khaled M. El-Asmar, Mohamed Abdel-Sattar, Ibrahim M. Abdelraheem, Ehab El-shafei

**Affiliations:** 1 Pediatric surgery unit, Helwan University; 2 Pediatric surgery department, Ain Shams University; 3 Pediatric surgery unit, Al Galaa Teaching Hospital

**Keywords:** Closed gastroschisis, Closing gastroschisis, Short bowel, Intestinal atresia

## Abstract

Closed gastroschisis is a rare entity usually associated with intestinal atresia and short bowel syndrome. We report two cases of closed gastroschisis presenting with neonatal intestinal obstruction and para-umbilical evisceration without an abdominal defect.

## INTRODUCTION

Gastroschisis is a congenital abdominal wall defect that is usually located on the right side of the umbilical cord, rarely; it is located in a mirror image position on its left side. Intestine, stomach, and occasionally other abdominal organs e.g. ovaries eviscerate through that defect [1]. In very rare cases, the defect itself closes around the eviscerated organs causing exit and/or entry level ischemia; a state which has been termed closed or closing gastroschisis and is representing around 6% of all cases of gastroschisis [2]. Sometimes the whole midgut is lost, a condition termed vanishing midgut [3].

## CASE SERIES

**Case 1:**

A full term 3 kg male presented with extra-abdominal bowel remnant just to the left side of a normally appearing umbilicus (Fig 1). Antenatal scan prior to delivery revealed intra-abdominal dilated bowel loops. Clinically, there was upper abdominal distension and bilious vomiting. Initial resuscitation was initiated. Abdominal X-ray showed suspicion of jejunal obstruction. Laboratory investigations were within normal limits. Echocardiography showed small patent foramen ovale. The baby was explored on day 2 via circum-umbilical incision. There was small bowel atresia. Remaining small bowel was 30 cm of blind-ended dilated proximal jejunum and 40 cm of small caliber distal ilium. Two fibrous strings were connecting the mesentery of both jejunum and ilium to an area in the inner aspect of the abdominal wall corresponding to the extra-abdominal bowel remnant. There was no abdominal wall defect. There was associated colonic atresia type IIIa at the level of transverse colon. The extra-abdominal bowel remnant and the connecting fibrous strings were excised. Short tapering of the proximal dilated jejunum, then primary end to end anastomosis was fashioned. Double barrel colostomy was done at the site of the colonic atresia leaving a length of 70 cm viable small bowel in addition to an intact ileocecal valve. Oral feeding was started on 10th postoperative day which was increased gradually to full feed on day 28. He was discharged on day 30.

**Figure:**
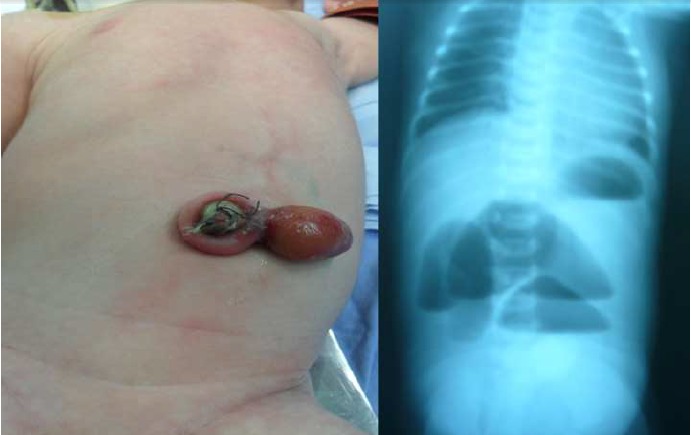
Figure 1: Extra-abdominal bowel remnant and x-ray showing small bowel obstruction.

**Case 2:**

A preterm (33weeks) male neonate weighing 1.9kg presented with extra-abdominal bowel remnant, 6 cm long, located to the right margin of an intact umbilical ring. Antenatal scan at 30 weeks of gestation showed dilated bowel loops. Follow-up scan performed 2 weeks later showed progressive dilatation of bowel loops without any abdominal wall defect. The baby developed neonatal intestinal obstruction. X-ray abdomen was suggestive of jejunal obstruction. On exploration, the jejunum was ending blindly 20 cm after the DJ junction. The eviscerated bowel remnant was connected from inside with the colon by a short fibrous stalk. There was 30 cm of non-atretic colon until the peritoneal reflection. Primary end-to end jejuno-colic anastomosis was performed. The patient was put on management for short bowel. One month later, the patient was re-explored due to partial intestinal obstruction. Second exploration revealed an ectatic poorly functioning jejunal loop. Side to side jejunocolic anastomosis was done for better bowel drainage. The baby died at the age of 4 months due to cholestatic liver failure and sepsis a complication of TPN.

## DISCUSSION

Spontaneous prenatal closure of the anterior abdominal wall defect in gastroschisis is very rare. Closure of the defect can be complete or incomplete with variable sequelae affecting bowel loops [4]. In cases of mild affection, there is a closing abdominal defect with viable bowel. Next in severity are cases with complete closure of the defect with a tiny stalk connecting the intra-abdominal bowel to an extracorporeal bowel remnant. These forms are termed closing gastroschisis and closed gastroschisis [2].

The sequence of events is not well known. It is not clear whether the closure of the defect is the primary event or occurs secondary to loss of the eviscerated bowel. Bhatia and colleagues in1996 proposed 3 scenarios [5]. All proposals attributed bowel atresia to vascular insult. First, abdominal wall defect seen early during pregnancy may close spontaneously around the herniated bowel and the supplying superior mesenteric artery resulting in strangulation and necrosis of the midgut. Second, the herniated fetal bowel may incarcerate within the defect leading to its ischemia and atrophy with subsequent closure of the defect. Third scenario starts by volvulus of the midgut with subsequent infarction, resorption, and closure of the defect.

Houben et al in 2009 clarified the criteria for successful antenatal ultrasonographic follow up in patients with gastroschisis [6]. They stated that after diagnosis of gastroschisis, fetal progress is monitored at 4 to 6 weeks intervals. More frequent scans were required in cases of intraabdominal bowel dilatation (>10 mm) or growth restriction of the fetus. The key for diagnosing closing gastroschisis is increased or persisted intraabdominal bowel dilatation with hyperperistalsis. At the same time, there is shrinkage or no increase in the size of extracorporeal bowel. After birth, they defined closing gastroschisis as circumferential or partial (>50%) closure of the ring around the protruding bowel associated with intestinal atresia, bowel ischemia, bowel necrosis, or viable intestine. No defect could be detected in our cases that could be explained by the delay in the initial ultrasound; however, progressive intraabdominal bowel dilatation was detected.

Infants suffering gastroschisis and vanishing midgut have nearly 70% mortality. Recently, marked improvement in the outcome has occurred due to advances in neonatal care and nutritional support and the introduction of home TPN regimens for pediatric age group [7]. Our survived case had adequate bowel length; 30 cm jejunum, 40 cm ilium, in addition to the viable part of the colon and ileocaecal valve. However, our second case succumbed to complications of management of short bowel syndrome.

## Footnotes

**Source of Support:** None

**Conflict of Interest:** None
